# Enhancing Adherence to Home-Based Expiratory Muscle Strength Training in Parkinson Disease: Randomized Controlled Trial of an mHealth Intervention

**DOI:** 10.2196/78022

**Published:** 2026-03-11

**Authors:** Martin Srp, Martina Hoskovcova, Rebeka Lagnerova, Katerina Dvorakova, Radim Kliment, Jan Muzik, Radim Krupicka, Ota Gal, Evzen Ruzicka

**Affiliations:** 1 Department of Neurology and Center of Clinical Neuroscience, General University Hospital and First Faculty of Medicine, Charles University, Katerinska 30, Prague, 120 00, Czech Republic, 420 224965550; 2 Department of Information and Communication Technologies in Medicine, Faculty of Biomedical Engineering, Czech Technical University in Prague, Prague, Czech Republic; 3 Department of Biomedical Informatics, Faculty of Biomedical Engineering, Czech Technical University in Prague, Kladno, Czech Republic

**Keywords:** mobile health, randomized controlled trial, Parkinson disease, expiratory muscle strength training, exercise adherence

## Abstract

**Background:**

Aspiration pneumonia is a leading cause of death in Parkinson disease (PD). Expiratory muscle strength training (EMST) is a promising intervention for respiratory and swallowing dysfunction. However, long-term EMST adherence is frequently poor in PD.

**Objective:**

This study aims to determine whether mobile health (mHealth)–assisted EMST with the *SpiroGym* app (Czech Technical University) improves long-term adherence and physiological outcomes versus conventional EMST among participants at risk for nonadherence.

**Methods:**

In this single-center, parallel, phase 2 randomized controlled trial, 75 individuals with PD were randomized 1:1 to conventional EMST (control; n=38) or the same protocol enhanced with the *SpiroGym* app (experimental; n=37), using a simple computer-generated randomization sequence. The *SpiroGym* is an mHealth app that provides real-time performance monitoring, direct visual feedback, and longitudinal progress tracking. All participants completed 8 weeks of semisupervised intensive EMST with biweekly in-person reassessments, followed by 16 weeks of unsupervised maintenance training. The primary outcome was adherence during weeks 8 to 24 among participants at risk for nonadherence, defined a priori at week 8 as Self-Efficacy for Home Exercise Program Scale (SEHEPS) less than 59. Because risk status was determined at week 8 and all participants subsequently entered the unsupervised phase, individuals not classified as at-risk were not excluded. Their data from week 8 onward were reported alongside the at-risk group. Secondary outcomes were changes in maximum expiratory pressure and SEHEPS.

**Results:**

No study-related adverse events occurred. Groups were well matched at baseline (control vs experimental: mean disease duration 7.0 (SD 5.7) vs 7.3 SD 4.7) y; mean Hoehn-Yahr 1.97 (SD 0.6) vs 2.0 (SD 0.5)). The mixed-effects model showed no significant 3-way interaction (group×interval×SEHEPS risk; *P*=.14). At week 24, the at-risk category for the nonadherence cohort comprised 34 participants (control, n=17; experimental, n=17). In this at-risk cohort, the experimental group demonstrated a smaller decline in adherence during weeks 8 to 24 than controls (*β*=496.9, 95% CI 130.7‐863.3; *P*=.008), completing 1073 (95% CI 643‐1502) expiratory maneuvers versus 525 (95% CI 358‐692). Maximum expiratory pressure increased in both groups from weeks 0 to 24, with larger gains in the experimental group (+43.1, 95% CI 32.4‐53.8 cmH_₂_O) than in controls (+22.8, 95% CI 13.8‐31.8 cmH_₂_O; *P*=.006; Cohen *d*=0.74). SEHEPS improved after intensive training in both groups, but only the experimental group exceeded the 12-point minimal detectable change at the 95% confidence limit.

**Conclusions:**

This is the first randomized controlled trial to integrate mHealth with EMST. Unlike prior studies in the EMST field, we focused on sustaining long-term exercise adherence. *SpiroGym*-assisted EMST resulted in higher long-term adherence and greater gains in expiratory muscle strength than conventional EMST. In real-world PD care, assessing self-efficacy after the supervised EMST phase may help identify individuals who would benefit from digital support, making mHealth-assisted EMST a practical approach for maintaining exercise adherence.

## Introduction

Neurodegenerative disorders are now the leading cause of disability worldwide [1]. Among them, Parkinson disease (PD) is the fastest-growing condition [2]. PD is a complex, progressive neurodegenerative disorder, with symptoms primarily arising from the loss of dopaminergic neurons in the brain [3]. Aspiration pneumonia is the leading cause of death in PD [4-6]. Among nonpharmacological interventions, expiratory muscle strength training (EMST) has shown promise in improving airway protection [7,8]. However, long-term adherence to EMST, which is critical to sustaining therapeutic benefits [9], is often poor in people with chronic conditions such as PD [10]. This challenge is compounded by the home-based, unsupervised nature of EMST, limiting opportunities for professional feedback and monitoring [11]. Additionally, dopaminergic dysfunction may reduce motivation, interest in pleasurable activities, and confidence in one’s ability to exercise, further potentiating nonadherence to EMST [12].

The widespread use of smartphones has enabled the development of health apps that track and manage symptoms, thereby strengthening self-care interventions for people with chronic illness [13-15]. Digital health technologies can expand access to home-based therapy and extend the benefits of in-person physiotherapy by providing real-time feedback, goal tracking, and performance summaries [14,16]. Feedback on past performance is particularly effective in strengthening exercise self-efficacy [17], which is defined as an individual’s belief in their ability to successfully perform goal-directed behaviors and stands out as a consistent predictor of exercise adherence across diverse populations [18-20], including people with PD [21]. Digital health features may be especially relevant for EMST, where patients may have limited insight into whether maneuvers are performed with adequate effort and technique, and where reinforcement is minimal when training alone at home.

To address barriers to long-term EMST, we developed the *SpiroGym* app using mobile health (mHealth) technologies to provide real-time performance monitoring, direct visual feedback, and longitudinal progress tracking. A pilot study [22] demonstrated the feasibility of *SpiroGym*-assisted EMST in PD. Just 2 weeks of training significantly improved maximum expiratory pressure (MEP) [22], achieving results comparable to longer EMST protocols [23,24]. These rapid gains were likely facilitated by increased training effort driven by real-time feedback. Further validation came from a recent multicenter study showing high usability and acceptance of *SpiroGym* across diverse international PD cohorts [25]. A subsequent proof-of-concept study reported excellent adherence during a 16-week unsupervised home-based EMST program using *SpiroGym*, even among participants unaccustomed to strength training [26]. That study also reported significant improvements in the Self-Efficacy for Home Exercise Program Scale (SEHEPS) [26]. Greater confidence on this scale correlated with long-term EMST adherence, highlighting self-efficacy’s critical role in sustained engagement [21,27,28]. While these findings underscore the potential of *SpiroGym*-assisted EMST, previous feasibility and proof-of-concept studies did not include an active-controlled randomized comparison against conventional EMST. Therefore, an active-controlled randomized controlled trial (RCT) is needed to determine whether mHealth-supported EMST improves adherence beyond conventional home-based EMST.

The primary objective of this RCT was to compare adherence during the unsupervised 16-week training period between conventional EMST and *SpiroGym*-assisted EMST in participants at risk for nonadherence. Secondary objectives were to evaluate changes in MEP and SEHEPS. Based on prior findings [22,26], we hypothesized that *SpiroGym*-assisted EMST would demonstrate superior results across these domains.

## Methods

### Study Design

We conducted a single-center, parallel, phase 2, randomized, active-controlled clinical trial that was retrospectively registered at ClinicalTrials.gov (NCT05728099) in 2023, after recruitment began in 2022 but before trial completion in 2025, and reported in accordance with the CONSORT-EHEALTH (Consolidated Standards of Reporting Trials of Electronic and Mobile Health Applications and Online Telehealth) guidelines (Checklist 1) [29]. The institutional review board (IRB; the ethics committee of the General University Hospital in Prague) confirmed that the protocol and study design reported in this paper are consistent with the study protocol and design that were reviewed and approved by the ethics committee before the trial started. A side-by-side comparison of key protocol elements across the IRB submission, the ClinicalTrials.gov record, and this paper is provided in Multimedia Appendix 1. This trial was conducted at the Department of Neurology, First Faculty of Medicine, Charles University and General University Hospital in Prague, Czech Republic, between March 2022 and January 2025. Participants were randomly assigned, with allocation concealment, to either the control group following the conventional EMST protocol or to the experimental group, following the same protocol enhanced with the *SpiroGym* app.

### Participants

Participants were recruited through convenience sampling from the Movement Disorders Center, Department of Neurology, First Faculty of Medicine, Charles University and General University Hospital in Prague, Czech Republic, between March 2022 and August 2024. The inclusion criteria were: (1) a diagnosis of PD confirmed by movement disorder specialist using the UK Brain Bank criteria [30], (2) a modified Hoehn and Yahr stage of I-IV in ON medication state, (3) an age of 40 to 80 years, and (4) stable dopaminergic medication (stable dose for at least 1 mo). The exclusion criteria were: (1) suspected parkinsonism due to causes other than idiopathic PD, (2) significant cognitive impairment (Montreal Cognitive Assessment score <19), (3) respiratory disorders or diseases, (4) current or previous history of head and neck cancer, (5) smoking within the past 5 years, (6) uncontrolled hypertension, and (7) previous experience with EMST.

### Randomization and Blinding

A simple computer-generated random allocation sequence (1:1 ratio) was generated by the trial statistician prior to study initiation using a web-based randomization tool. All outcome assessors and data analysts were blinded to group allocation. However, the physiotherapists overseeing the EMST training could not be blinded, as the use of the *SpiroGym* app in the experimental group was apparent. Participants were informed that the study compared 2 EMST programs. They were not given specific details regarding the nature of each program or the hypothesis that one approach might be superior, thereby maintaining participant blinding as effectively as possible under these circumstances.

### Study Outcome Parameters

The primary aim of the study was to compare EMST adherence during the unsupervised home-training phase (weeks 8‐24) between a control group and an experimental group among patients at risk for nonadherence, defined as a SEHEPS score below 59 points at the 8-week visit. The decision to identify at-risk participants at week 8 was based on previous research [26], demonstrating no correlation between baseline SEHEPS scores and later adherence, indicating that week 8 is a more reliable point to detect adherence risk. Because the SEHEPS classification was obtained at week 8, randomization at baseline could not be stratified by SEHEPS. Allocation was not altered thereafter, and participants remained in their originally randomized arms. Secondary outcomes included changes in MEP after the intensive EMST phase (weeks 0‐8) and the maintenance phase (weeks 8‐24), as well as changes in SEHEPS scores following the intensive phase (weeks 0‐8). Since the risk of nonadherence was defined at week 8, and all participants proceeded from that visit into the unsupervised phase, we did not exclude those classified as not at risk. Their data from week 8 onward are reported alongside the at-risk group to provide a comprehensive view.

### Assessment Visits

The same assessment protocol was completed at pre- and posttreatment visits. Outcome examinations were carried out at the same time of day in the ON medication state (1 h after regular dopaminergic medication) at the Department of Neurology, First Faculty of Medicine, Charles University and General University Hospital in Prague, Czech Republic.

### Adherence (Primary Outcome)

Adherence during the unsupervised home-training phase (weeks 8‐24) was defined as the cumulative number of completed expiratory maneuvers. The prescribed total was 800 maneuvers. In the experimental group, adherence was monitored using the *SpiroGym* app, which automatically recorded the number of completed EMST maneuvers. In the control group, adherence was tracked via self-reported training diaries (Multimedia Appendix 2), where participants recorded the number of EMST maneuvers completed.

### MEP and SEHEPS (Secondary Outcomes)

MEP assessments were performed using a flanged rubber mouthpiece connected to a pressure manometer (Micro RPM, Vyaire Medical). The procedure followed standards for respiratory muscle testing [31]. Seated upright, participants inhaled to total lung capacity and then exhaled forcefully into the manometer. Participants received verbal encouragement throughout the MEP assessment. Nose clips were applied to prevent air leakage through the nose. Participants were explicitly instructed to support their cheeks and lips with their hands during the MEP maneuver, as any cheek bulging and lip leakage can dissipate pressure and result in artificially reduced values [32]. One week before baseline testing, a training MEP session was conducted to minimize learning effects [33]. Before each formal assessment, participants completed a warm-up of 6 MEP maneuvers at 50% maximal effort [34] to account for neural facilitation from repeated efforts [33]. At least 3 measurements with a less than 10% variation between them were taken, and the highest value (cmH_2_O) was recorded as the MEP.

The exercise self-efficacy was assessed using the SEHEPS [35]. The test-retest reliability of SEHEPS has been demonstrated as excellent with an intraclass correlation coefficient (ICC) of 0.96 (95% CI 0.91‐0.98) in patients with PD [26]. The minimal detectable change at the 95% confidence limit (MDC_95_) for SEHEPS is 12 points [35]. A total self-efficacy score is derived by summing responses to 12 items, resulting in a range from 0 to 72, where higher scores indicate greater exercise self-efficacy. A SEHEPS score of less than 59 points identifies individuals at risk of nonadherence (defined as <70% of exercise adherence) to home exercise programs [35]. At baseline, patients completed the SEHEPS based on their confidence in undertaking any long-term home exercise program. At week 8, they completed the SEHEPS again, focusing specifically on their confidence in continuing long-term EMST.

### Training Protocols

#### Conventional EMST

Patients performed EMST at home using an expiratory muscle trainer (the EMST150; Aspire Products, LLC). During the intensive phase, therapy sessions consisted of 5 sets of 5 forceful expirations, completed on 5 self-selected days per week. The EMST150 was set at 75% of each participant’s MEP value. If 75% was not initially achievable, participants were instructed to temporarily lower the threshold to a tolerable level and progressively readjust it upward across subsequent sessions, with the aim of reaching 75% as soon as feasible. Participants were instructed to occlude their nose with nose clips, take a big breath in, and blow as forcefully as possible into the device to open the valve. The expiratory effort should last a couple of seconds for the air to move through the device. A typical training session lasted approximately 10 minutes. Every 2 weeks during the intensive phase, participants attended in-person visits. At each visit, a physiotherapist reassessed MEP, readjusted the device to 75% of the participant’s current MEP, and briefly verified technique (1 set of 5 expiratory maneuvers). A full 25-repetition training session was not conducted. The same procedure was applied in both groups (control and experimental). After the initial 8-week period, patients continued EMST for an additional 16 weeks (maintenance phase) without scheduled visits. In the maintenance phase, the prescription was 5 sets of 5 forceful expirations twice weekly, with optional extra sessions to mirror real-world practice. In our clinical experience, some patients prefer to exercise more often than twice a week. During the maintenance phase, participants were instructed to maintain the threshold set at the week-8 visit. Self-adjustment was not permitted. If they felt they could tolerate a higher resistance or if resistance needed to be decreased (eg, after an intercurrent illness), they were instructed to contact the study team for an in-person visit to reassess MEP and have the device reset by the physiotherapist. The same guidance applied to both groups. This protocol was adapted from our earlier proof-of-concept study [26].

#### SpiroGym-Assisted EMST

Participants in the experimental group followed the same EMST schedule and intensity as the control group, but their EMST150 was coupled with the *SpiroGym* app on a study smartphone (Samsung Galaxy A20e) preloaded with the app. The app monitors expiratory training in real time by analyzing acoustic signals captured via an external microphone mounted to the EMST device using a custom 3D-printed ring fabricated by our team. A download link to the ring’s build instructions and a photo of the correct attachment on the EMST150 are available in [36]. The attachment does not alter resistance, airflow, or the valve and standardizes the microphone’s position and angle. The microphone was wired to the phone via a 3.5-mm jack. When the training is performed correctly, the trainer’s valve opens, generating increased airflow and sound. These signals are converted by the app into a visual feedback curve displayed on the participant’s smartphone (Multimedia Appendix 3). Additionally, the app automatically records session data, creating a digital training diary that enables patients to review their performance over time. Although *SpiroGym* can send exercise reminders, this feature was not enabled in the present trial because participants were loaned a study device and were unlikely to carry it during the day, making push notifications unreliable. Technical details of the app have been described in detail elsewhere [22].

### Sample Size

Sample size calculations were based on the primary outcome (adherence to unsupervised EMST). Because no prior controlled studies had compared EMST alone with *SpiroGym*-assisted EMST, we estimated the effect size from an internal pilot study. We analyzed week 8 to 24 adherence in the first 10 PD participants in each arm with SEHEPS less than 59 at week 8. Their adherence data yielded a Cohen *d* of 0.92 (unpublished internal data). A power analysis using G*Power (version 3.1.9.3; Heinrich Heine University Düsseldorf) [37] indicated that 16 participants at risk for nonadherence per group would achieve 80% power at a 1-sided 5% type 1 error rate to detect this effect size. Accounting for a 20% attrition rate, each group was thus targeted to include 19 participants.

### Safety

Safety was assessed by monitoring adverse events throughout the study. During the intensive phase, safety was monitored via bi-weekly in-person visits. During the maintenance phase, participants were instructed to contact the study team by phone if any adverse effects occurred.

### Statistical Analysis

Baseline variables were tested for normality (Kolmogorov-Smirnov test). Normally distributed data were compared with independent 2-tailed *t* tests; nonnormal data with the Mann-Whitney *U* test. Sex distribution was analyzed by the Fisher exact test. A linear mixed-effects model was used to analyze adherence across time intervals, groups, and SEHEPS subgroups, accounting for repeated measures within individuals. The model formula was:


adherence∼group×interval×SEHEPSgroup+(1∣ID)

where group (control/experimental), interval (wk 0-8/8-24), and SEHEPS group (<59/≥59) were included as fixed effects and their interactions, and participant ID was included as a random intercept to account for within-individual correlation. Model assumptions were checked using residual diagnostics (Shapiro-Wilk and Breusch-Pagan tests). Post hoc analyses were performed to assess group differences at each interval. Adherence was compared between groups for weeks 0 to 8 and 8 to 24, and within SEHEPS subgroups (<59 vs ≥59) using the Mann-Whitney *U* test. Four-week interval (8-12, 12-16, 16-20, and 20-24) adherences were also analyzed using the same approach. MEP changes (0‐8, 8‐24, and 0‐24 wk) were assessed with paired *t* tests within groups and independent *t* tests between groups. Effect size was expressed as Cohen *d* (95 % CI), interpreted as moderate (≥0.5) and large (≥0.8). Associations between adherence and SEHEPS were examined with Spearman ρ. Our sample-size planning used a 1-sided hypothesis based on prior evidence [26] supporting a directional expectation (*SpiroGym* >control). However, for the inferential analyses, we adopted 2-sided tests to provide a more conservative interpretation. Missing data were examined for randomness using the Little Missing Completely at Random test to determine whether the missingness was random or systematic. The missing data originated from participants who did not complete the therapy. *P*<.05 was considered statistically significant. All analyses were performed in Python (version 3.12; Python Software Foundation) using the *scipy* and *statsmodels* packages.

### Ethical Considerations

This study was conducted in accordance with the principles of the Declaration of Helsinki. The study was approved prospectively by the IRB of the General University Hospital in Prague (protocol 223/20 S-IV). All participants provided written informed consent prior to any study procedures, including consent for collection, analysis, and publication of deidentified data. Participation was entirely voluntary, and participants could withdraw at any time without consequence. Participants did not receive any financial compensation. All data were deidentified before analysis and stored in password-protected files accessible only to the research team in accordance with institutional data protection policies. The paper and supplementary materials contained no identifiable images of participants. However, Multimedia Appendix 3 shows an identifiable study researcher (not a participant). Written consent for publication from this individual has been obtained.

## Results

### Participants

**Figure 1.**
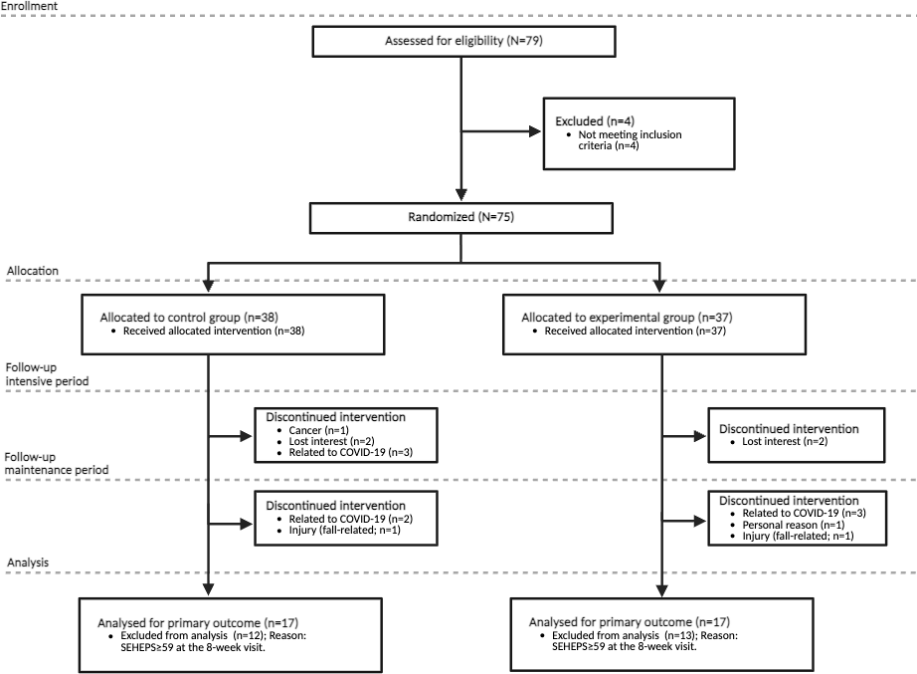
Flowchart of the patients with Parkinson disease in the study. SEHEPS: Self-Efficacy for Home Exercise Program Scale.

No study-related adverse events occurred during the study period. To achieve the target of 19 participants with PD in each arm with low self-efficacy, recruitment continued until this quota was achieved, which did not occur until enrolling 75 individuals. Patients in the control group (n=38) and *SpiroGym*-assisted EMST group (n=37) were well-matched at baseline. Baseline demographics also did not differ significantly between the at-risk (SEHEPS <59) and not-at-risk (SEHEPS ≥59) subgroups. A summary of demographic and clinical characteristics is provided in Table 1. A total of nine participants from the control group and seven participants from the experimental group did not complete the study (detailed reasons are provided in Figure 1), resulting in missing data. The Little Missing Completely at Random test was nonsignificant (*P*>.05), indicating that the data were missing completely at random. Over the 24-week period, the mean MDS-UPDRS-III motor score increased by 1.8 (SD 5.2) points in the experimental group and by 1.7 (SD 3.9) points in the control group, with no significant difference between the groups (*P*=.95).

**Table 1.** Baseline demographic characteristics for all randomized participants and subgroups defined at week 8 by risk for nonadherence using the Self-Efficacy for Home Exercise Program Scale (SEHEPS): at-risk (SEHEPS <59) and not at-risk (SEHEPS ≥59).

Measure	All patients			At-risk patients, SEHEPS <59			Not-at-risk patients, SEHEPS ≥59		
	Control group	Experimental group	*P* value	Control group	Experimental group	*P* value	Control group	Experimental group	*P* value
Sex (male/female), n	21/17	24/13	.48	10/8	10/9	>.99	7/6	13/3	.15
Age (y), mean (SD)	65.4 (9.5)	65.5 (8.9)	.99	66.2 (9.0)	64.8 (8.4)	.62	67.8 (9.8)	66.2 (9.6)	.68
Height (cm), mean (SD)	174 (8.9)	173 (9.3)	.68	174.8 (8.8)	171.0 (9.1)	.21	171.8 (8.4)	175.2 (9.0)	.31
Weight (kg), mean (SD)	78.3 (19.2)	79.8 (18.5)	.74	76.2 (17.0)	79.0 (17.7)	.63	78.9 (17.5)	82.6 (20.2)	.61
BMI, mean (SD)	25.8 (5.0)	26.5 (4.4)	.51	24.8 (4.4)	26.7 (4.0)	.17	26.7 (5.4)	26.7 (4.8)	.99
Education (y), mean (SD)	14.9 (2.9)	15.6 (2.9)	.25	15.6 (3.0)	15.1 (2.7)	.60	14.3 (2.6)	15.8 (2.7)	.10
Disease duration (y), mean (SD)	7 (5.7)	7.3 (4.7)	.58	6.2 (6.2)	7.5 (4.5)	.49	6.9 (4.3)	7.6 (5.1)	.72
Hoehn-Yahr, mean (SD)	1.97 (0.6)	2 (0.5)	.84	2.0 (0.5)	2.1 (0.3)	.46	2.2 (0.6)	1.9 (0.7)	.38
MDS-UPDRS III^a^ (ON), mean (SD)	17.1 (8.8)	17.5 (9.5)	.86	17.3 (9.3)	20.2 (7.5)	.30	17.2 (9.4)	15.1 (11.2)	.61
MoCa^b^, mean (SD)	26.3 (3.0)	25.9 (2.9)	.64	25.7 (3.2)	26.3 (2.4)	.56	27.4 (3.1)	25.5 (3.3)	.13
LEDD^c^ (mg/d), mean (SD)	672 (300)	696 (295)	.75	690.0 (313.6)	673.2 (347.9)	.89	697 (280.0)	726.4 (253.0)	.78
SEHEPS, mean (SD)	40.7 (15.4)	39.8 (17.3)	.81	32.8 (12.3)	30.0 (13.8)	.52	48.3 (16.5)	49.6 (14.6)	.83
Maximum expiratory pressure (cmH_2_O), mean (SD)	130 (50)	137 (42.9)	.56	133.5 (44.7)	130.9 (45.8)	.86	128.7 (54.5)	141.4 (41.1)	.48

a MDS-UPDRS III: Movement Disorder Society—Unified Parkinson’s Disease Rating Scale, Part III: Motor Examination

b MoCA: Montreal Cognitive Assessment.

c LEDD: levodopa equivalent daily dose.

### Primary Outcome: Adherence

The primary analysis using a linear mixed-effects model did not show a significant 3-way interaction between group, time interval, and baseline risk for nonadherence (group×interval×SEHEPS group; *P*=.14; Multimedia Appendix 4). However, a planned secondary analysis of participants at high risk for nonadherence (SEHEPS <59 at week 8) revealed a significant group×interval interaction. In this subgroup, the intervention group showed a significantly smaller decline in adherence during weeks 8 to 24 compared to the control group, as confirmed by a post hoc test (*β*=496.9, 95% CI 130.7-863.3; *P*=.008), indicating that the intervention was effective in sustaining adherence specifically in this at-risk population.

### Post Hoc Analyses: Adherence

**Figure 2.**
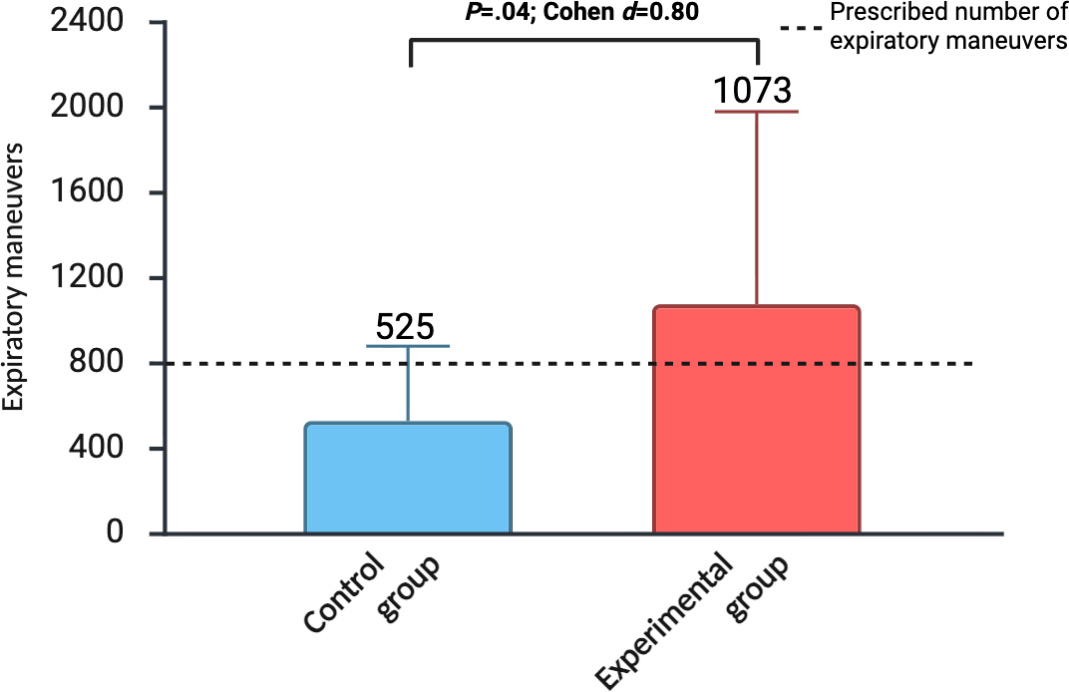
Adherence to expiratory muscle strength training during weeks 8 to 24 in participants at risk for nonadherence based on the Self-Efficacy for Home Exercise Program Scale (SEHEPS <59). The values are presented as mean (SD).

**Figure 3.**
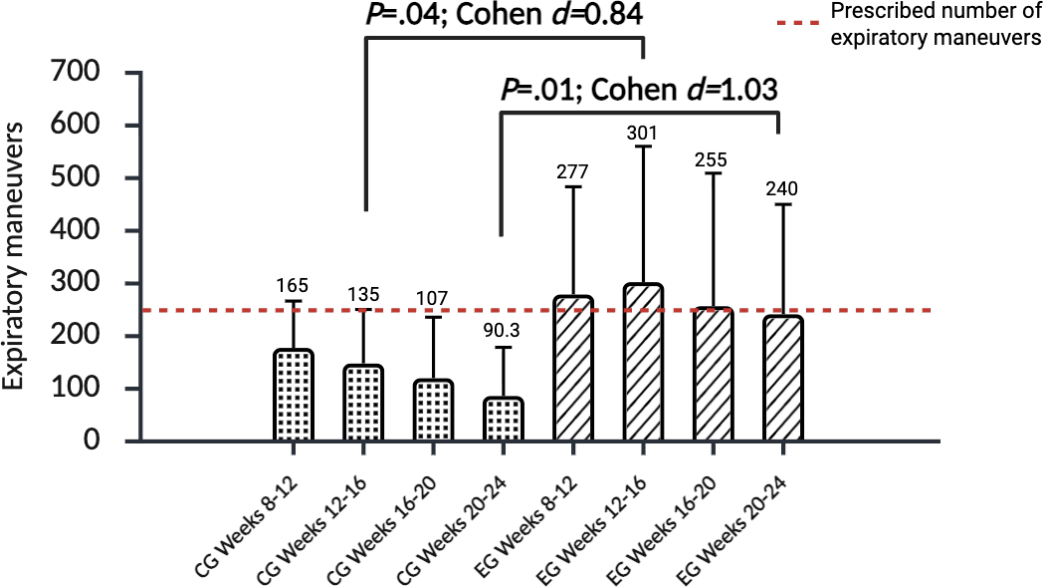
Four-week interval adherence to expiratory muscle strength training during weeks 8 to 24 in patients at risk for nonadherence (Self-Efficacy for Home Exercise Program Scale [SEHEPS] <59). The values are presented as mean (SD). CG: control group; EG: experimental group.

Among participants at risk for nonadherence (n=34), adherence during the maintenance phase (wk 8‐24) was significantly higher in the experimental group than in the control group (*P*=.04; Cohen *d*=0.80; Figure 2 and Table 2). Within this group, 47% (8/17) of *SpiroGym* participants versus 18% (3/17) of controls achieved the minimum prescribed 800 or more repetitions during weeks 8 to 24. A 4-week interval analysis revealed that between-group differences in adherence reached significance during weeks 12 to 16 (*P*=.04; Cohen *d*=0.84) and weeks 20 to 24 (*P*=.01; Cohen *d*=1.03), with no significant differences during weeks 8 to 12 or 16 to 20 (Figure 3). Among patients (n=25) without risk for nonadherence, no difference in adherence was observed during the maintenance period between the control and experimental groups (*P*=.34; Cohen *d*=0.22; Table 2; Multimedia Appendix 5). For the overall sample (n=59), adherence during the intensive phase (wk 0‐8) did not differ significantly between groups (*P*=.05; Cohen *d*=0.49), while during the maintenance phase (wk 8‐24), the experimental group achieved significantly greater adherence (*P*=.02; Cohen *d*=0.58; Table 2).

**Table 2.** Adherence to expiratory muscle strength training by study interval: weeks 0 to 8 (intensive phase) and weeks 8 to 24 (maintenance), for the full sample and for subgroups defined at week 8 by Self-Efficacy for Home Exercise Program Scale (SEHEPS) risk (at-risk: SEHEPS <59; not at-risk: SEHEPS ≥59).

Time interval and group		Participants, n	Repetitions, mean (SD)		95% CI	Between-group effect	
						*P* value	Cohen *d* (95% CI)
Overall adherence (all patients)							
Weeks 0-8						.05	0.49 (0.01 to 0.97)
CG^a^		32	953 (83.2)		924 to 982		
EG^b^		35	998 (95.7)		966 to 1030		
Weeks 8-24						.02	0.58 (0.06 to 1.10)
CG		29	771 (538)		575 to 967		
EG		30	1139 (712)		884 to 1393		
Adherence analysis based on SEHEPS at week 8							
Weeks 8-24						.04	0.80 (0.10 to 1.50)
CG SEHEPS <59		17	525 (351)		358 to 692		
EG SEHEPS <59		17	1073 (903)		643 to 1502		
Weeks 8-24						.34	0.22 (–0.57 to 1.0)
CG SEHEPS ≥59		12	1120 (577)		794 to 1446		
EG SEHEPS ≥59		13	1225 (352)		1034 to 1416		
Four-week interval adherence analysis in patients at risk of nonadherence							
Weeks 8-12						.15	0.74 (–0.01 to 1.4)
CG		17	165 (98.4)		118 to 212		
EG		17	277 (204)		180 to 374		
Weeks 12-16						.04	0.84 (0.14 to 1.54)
CG		17	135 (108)		84 to 186		
EG		17	301 (258)		178 to 423		
Weeks 16-20						.06	0.75 (0.05 to 1.45)
CG		17	107 (118)		51 to 163		
EG		17	255 (252)		135 to 375		
Weeks 20-24						.01	1.03 (0.32 to 1.75)
CG		17	74 (90.3)		31 to 116		
EG		17	240 (208)		141 to 339		

a CG: control group.

b EG: experimental group.

### Secondary Outcomes

#### Maximum Expiratory Pressure

From baseline to week 8, MEP increased by 28.4 cmH_₂_O in the control group (*P*<.001; Cohen *d*=0.58) and by 42.3 cmH_₂_O in the experimental group (*P*<.001; Cohen *d*=0.89), with the experimental group showing significantly greater improvement (*P*=.03; Cohen *d*=0.54). During the unsupervised period (wk 8‐24), MEP decreased by 6.8 cmH₂O in the control group (*P*=.11; Cohen *d*=–0.15) and increased by 0.1 cmH_₂_O in the experimental group (*P*=0.96, Cohen *d*=0.004), with no significant between-group difference (*P*=.16; Cohen *d*=0.37). Over the entire 24-week study period, MEP increased by 22.8 cmH_₂_O in the control group (*P*<.001; Cohen *d*=0.50) and by 43.1 cmH_₂_O in the experimental group (*P*<.001; Cohen *d*=0.91), with the experimental group demonstrating significantly greater improvements (*P*=.006; Cohen *d*=0.74). Table 3 summarizes MEP outcomes.

**Table 3.** Overall maximum expiratory pressure changes by study interval: weeks 0 to 8 (intensive phase) and weeks 8 to 24 (maintenance), for the full sample and for subgroups defined at week 8 by Self-Efficacy for Home Exercise Program Scale (SEHEPS) risk (at-risk: SEHEPS <59; not at-risk: SEHEPS ≥59).

Time interval and group		Participants (n)	Preinterval, mean (SD)	Postinterval, mean (SD)	Within-group change		Between-group effect	
					Mean (SD) Δ^a^	95% CI Δ	*P* value	Cohen *d* (95% CI)
All patients								
Weeks 0-8							.03	0.54 (0.05 to 1.03)
CG^b^		31	131.5 (48.2)	159.9 (50.3)	+28.4 (25.8)	19.3 to 37.5		
EG^c^		35	135.7 (43.4)	178.1 (51.7)	+42.3 (25.9)	33.8 to 50.9		
Weeks 8-24							.16	0.37 (–0.14 to 0.89)
CG		29	161.3 (50.4)	154.6 (42.6)	–6.8 (22.4)	–15 to 1.4		
EG		30	179.7 (51.1)	179.9 (52.8)	+0.1 (13.7)	–4.8 to 5.0		
Weeks 0-24							.006	0.74 (0.21 to 1.27)
CG		29	131.8 (48.1)	154.6 (42.6)	+22.8 (24.7)	13.8 to 31.8		
EG		30	136.8 (41.4)	179.9 (52.8)	+43.1 (29.9)	32.4 to 53.8		
Subgroup analysis based on exercise self-efficacy (SEHEPS at wk 8)								
Weeks 8-24 (SEHEPS <59)							.04	0.73 (0.04 to 1.43)
CG		17	168.1 (53.4)	153.5 (45.4)	–14.6 (21)	–24.6 to –4.6		
EG		17	180.1 (49.9)	177.9 (50.7)	–2.2 (11.3)	–7.6 to 3.1		
Weeks 8-24 (SEHEPS ≥59)							.90	–0.06 (–0.84 to 0.73)
CG		12	151.8 (46.4)	156.1 (40)	+4.2 (20.4)	–7.3 to 15.8		
EG		13	179.2 (54.5)	182.5 (57.5)	+3.2 (16.3)	–5.6 to 12.1		

a Δ: change.

b CG: control group.

c EG: experimental group.

#### Self-Efficacy for Home Exercise Program Scale

From baseline to week 8, the mean SEHEPS score significantly increased by 11.2 (SD 13.6, 95% CI 6.36-15.96; *P*<.001) points in the control group and by 14.5 (SD 14.0, 95% CI 9.91-19.2; *P*<.001) points in the experimental group. Furthermore, at week 8, SEHEPS scores correlated positively with adherence during weeks 8 to 24 in both groups (control: ρ=0.60, *P*=.001; experimental: ρ=0.44, *P*=.01).

## Discussion

### Principal Findings

This phase 2 RCT compared the efficacy of a conventional EMST protocol with a *SpiroGym*-assisted protocol. For the primary objective, participants at risk for EMST nonadherence who trained with *SpiroGym* sustained greater adherence during the unsupervised phase than those following conventional EMST. Regarding secondary objectives, the *SpiroGym* group achieved greater improvements in MEP and a clinically meaningful increase in self-efficacy, whereas the control group resulted in smaller but still significant gains.

Building on earlier findings [26] demonstrating sustained user engagement with the *SpiroGym* app during long-term EMST, our study adds further evidence that *SpiroGym* can effectively support adherence over time. Notably, participants with sufficient self-efficacy (SEHEPS ≥59) performed 30% more expiratory maneuvers than the minimum prescribed target during the maintenance period in both groups. In contrast, among those with low self-efficacy (SEHEPS <59), adherence in the control group dropped from 83% (wk 8‐12) to 37% (wk 20‐24), while the *SpiroGym* group consistently (every 4-wk interval) maintained adherence 20% above the prescribed maneuver target. These findings align with Chung et al [38], who reported higher adherence and enhanced self-efficacy in an mHealth-supported exercise program compared to a paper-based approach. Similarly, Sørensen et al [39] reported that feedback-based inspiratory muscle training yielded better adherence than self-reported protocols over a 12-week unsupervised period.

In line with our prior study [26], we observed a positive correlation between SEHEPS scores at week 8 and adherence during weeks 8 to 24 in both groups, suggesting that self-efficacy assessed after the intensive phase may predict long-term EMST compliance. Although SEHEPS scores improved significantly in both protocols, only the *SpiroGym* group exceeded the 12-point MDC_95_ threshold [35], indicating a true increase in exercise self-efficacy beyond measurement error—a finding supported by previous research [40].

These findings have direct clinical implications for tailoring EMST. Patients should be assessed for self-efficacy following the intensive phase. Those with low self-efficacy may benefit from enhanced supervision or motivational support, such as the *SpiroGym* app, to sustain adherence during the maintenance period. Conversely, conventional EMST may be sufficient for patients with higher self-efficacy. Beyond self-efficacy, other factors such as apathy or caregiver support may plausibly influence adherence and treatment outcomes and may guide treatment selection in practice [41,42]. Screening for these factors alongside SEHEPS may help triage patients to conventional versus supported EMST.

Another key finding relates to real-time visual feedback and its impact on MEP. After the 8-week intensive phase, both groups achieved meaningful MEP gains, but the increase was significantly larger in the *SpiroGym* group (*P*=.03; Cohen *d*=0.54). On average, MEP increased by 22% in the control group and 31% in the experimental group, consistent with prior reports of approximately 24% MEP gains in PD [8]. Based on the observation that, during weeks 0‐8, the experimental group showed greater MEP gains despite comparable adherence, we hypothesize that real-time visual feedback may enhance motivational aspects of learning and thereby facilitate better skill acquisition. This interpretation is consistent with prior studies showing amplified respiratory muscle activation under visual feedback conditions [43]. Given the role of EMST in airway protection [4,6], these superior MEP outcomes suggest that SpiroGym-assisted EMST may translate into improved clinical outcomes for individuals with dysphagia or impaired cough, although this remains to be confirmed in future research.

A further notable result pertains to long-term MEP maintenance in patients at risk for nonadherence (SEHEPS <59). While the control group showed an average 9% decline in MEP between weeks 8 and 24, the *SpiroGym* group maintained stable values. Previous studies report detraining-related MEP declines of 2% to 20% over 1 to 18 months in PD [9,44,45]. Long-term maintenance training at least twice per week is generally recommended [46]. In our study, control group participants missed an average of 11 out of 32 scheduled sessions, falling below the minimum recommended dose and likely contributing to the observed MEP reduction. In contrast, adherence in the experimental group was sufficient to prevent detraining effects. On the other hand, resistance-training adaptations typically extend beyond 8 weeks in both healthy adults and people with PD. One likely explanation for the plateau in MEP gains is that no participant requested recalibration during the maintenance period. As a result, training thresholds remained fixed, reducing progressive resistance. This contrasts with evidence from a 2-year randomized trial, in which Corcos et al [47] showed that people with PD who trained twice weekly with progressively increased resistance continued to improve upper-limb strength up to the 24-month visit, whereas those following a nonprogressive program did not. A second contributor may be the device load ceiling. In a subset of experimental participants (n=11), week-8 MEP exceeded 200 cmH_₂_O, placing the 75% training target above the EMST150’s maximum load. To sustain MEP progression, future protocols should implement routine recalibration during the maintenance period and, for participants approaching the ceiling, employ devices with a higher resistance range.

### Limitations

This study has several limitations. First, adherence in the control group was based on self-reported training diaries, which might be prone to inaccuracy [48]. Second, we did not include objective measures of swallowing function or cough efficacy, limiting conclusions about the broader clinical impact of the observed increase in the MEP. Third, the sample primarily consisted of individuals with mild-to-moderate PD, which limits the generalizability of findings to those with more advanced PD or cognitive impairment. Fourth, we did not collect person-centered outcomes (eg, treatment burden and quality of life). Although a separate study reports acceptability of *SpiroGym*, it does not provide a head-to-head comparison with conventional EMST [25,26]. Consequently, it remains unclear whether SpiroGym-supported EMST reduces perceived burden or improves quality of life relative to standard care. Fifth, during the maintenance phase, participants were instructed to perform five sets of five expirations twice weekly, with the option to train more frequently to reflect real-world practice. This flexibility allowed some individuals to accumulate higher totals of expiratory maneuvers, which may have influenced the effect size.

Future studies should involve larger cohorts, longer follow-up periods, and objective assessments of airway protection. In addition, qualitative and mixed methods studies comparing *SpiroGym* with traditional EMST are warranted to understand patient experience (eg, satisfaction, motivation, enjoyment, and usability).

To support further research and clinical adoption, the *SpiroGym* app is freely available at [36], along with detailed hardware specifications. A user manual is included in Multimedia Appendix 6.

### Conclusions

This is the first RCT to integrate mHealth with EMST. Unlike prior studies in the EMST field, we focused on long-term exercise adherence, which is critical for sustaining therapeutic effects. Compared with conventional EMST, *SpiroGym*-assisted training led to higher adherence, most notably among patients prone to nonadherence, and produced greater expiratory muscle strength gains. In routine PD care, evaluating self-efficacy after the supervised EMST phase may help identify patients who are likely to benefit from digital support, positioning mHealth-assisted EMST as a practical strategy to sustain exercise adherence.

## Supplementary material

10.2196/78022 Multimedia Appendix 1 A side-by-side comparison of key protocol elements across the institutional review board submission, the ClinicalTrials.gov record (NCT05728099), and published manuscript.

10.2196/78022 Multimedia Appendix 2 Self-monitoring training diaries.

10.2196/78022 Multimedia Appendix 3 Visual feedback provided by the SpiroGym app during expiratory muscle strength training.

10.2196/78022 Multimedia Appendix 4 Linear mixed-effects model for adherence.

10.2196/78022 Multimedia Appendix 5 Four-week interval adherence analysis in patients without risk of nonadherence.

10.2196/78022 Multimedia Appendix 6 SpiroGym user manual.

10.2196/78022 Checklist 1 CONSORT-EHEALTH checklist.

## Abbreviations

CONSORT-EHEALTH: Consolidated Standards of Reporting Trials of Electronic and Mobile Health Applications and Online Telehealth
EMST: expiratory muscle strength training
ICC: intraclass correlation coefficient
IRB: institutional review board
MDC95: minimal detectable change at the 95% confidence level
MEP: maximum expiratory pressure
mHealth: mobile health
PD: Parkinson disease
RCT: randomized controlled trial
SEHEPS: Self-Efficacy for Home Exercise Program Scale
