# Supplementary material for: Enhancing Adherence to Home-Based Expiratory Muscle Strength Training in Parkinson Disease: Randomized Controlled Trial of an mHealth Intervention
Source: J Med Internet Res. 2026 Mar 11;28:e78022. doi: 10.2196/78022 (PMC12978541; doi:10.2196/78022)
Supplement: Multimedia Appendix 1 [file jmir-v28-e78022-s001.docx]

| **Key protocol elements** | **Protocol submitted to the institutional review board** | **ClinicalTrial.gov**  **registration** | **Published manuscript** |
| --- | --- | --- | --- |
| **Study design** | Randomized, controlled, two-arm study, blinded, single-center. | Randomized, controlled, two-arm study, blinded, single-center. | Randomized, controlled, two-arm study, blinded, single-center. |
| **Control group program** | EMST150 device; training recorded in a paper diary. | EMST150 device; training recorded in a paper diary. | EMST150 device; training recorded in a paper diary. |
| **Experimental group program** | EMST150 device + SpiroGym mobile application. | EMST150 device + SpiroGym mobile application. | EMST150 device + SpiroGym mobile application. |
| **Duration, training intensity, and level of supervision – intensive phase (week 0–8)** | 8 weeks: 5 days/week, 5 sets × 5 forceful expirations; resistance set to ~75% of MEP; semi-supervised training. | 8 weeks: 5 days/week, 5 sets × 5 forceful expirations; resistance set to ~75% of MEP; semi-supervised training. | 8 weeks: 5 days/week, 5 sets × 5 forceful expirations; resistance set to ~75% of MEP; semi-supervised training. |
| **Duration, training intensity, and level of supervision – maintenance phase (week 8–24)** | 16 weeks: at least twice per week, 5 sets × 5 expirations; unsupervised training. | 16 weeks: at least twice per week, 5 sets × 5 expirations; unsupervised training. | 16 weeks: at least twice per week, 5 sets × 5 expirations; unsupervised training. |
| **Primary study objective** | Long-term adherence and compliance with respiratory training, assessed during the maintenance phase (weeks 8–24) in patients with Parkinson’s disease. | Long-term adherence and compliance with respiratory training, assessed during the maintenance phase (weeks 8–24) in patients with Parkinson’s disease. | Long-term adherence and compliance with respiratory training, assessed during the maintenance phase (weeks 8–24) in patients with Parkinson’s disease. |

**Supplementary Table 1.** A side-by-side comparison of key protocol elements across the institutional review board submission, the ClinicalTrials.gov record (NCT05728099), and published manuscript
