# Supplementary material for: Enhancing Adherence to Home-Based Expiratory Muscle Strength Training in Parkinson Disease: Randomized Controlled Trial of an mHealth Intervention
Source: J Med Internet Res. 2026 Mar 11;28:e78022. doi: 10.2196/78022 (PMC12978541; doi:10.2196/78022)
Supplement: Multimedia Appendix 2 [file jmir-v28-e78022-s002.docx]

**Supplementary Table 2** Self-monitoring training diaries

| Patients ID: | | | | | |
| --- | --- | --- | --- | --- | --- |
| **Were all 25 repetitions completed?** | | | | | |
| **Date ®** |  |  |  |  |  |
| Circle the appropriate answer | YES/NO | YES/NO | YES/NO | YES/NO | YES/NO |
| If NO, how many reps? |  |  |  |  |  |
|  | | | | | |
| **Date ®** |  |  |  |  |  |
| Circle the appropriate answer | YES/NO | YES/NO | YES/NO | YES/NO | YES/NO |
| If NO, how many reps? |  |  |  |  |  |
|  | | | | | |
| **Date ®** |  |  |  |  |  |
| Circle the appropriate answer | YES/NO | YES/NO | YES/NO | YES/NO | YES/NO |
| If NO, how many reps? |  |  |  |  |  |
|  | | | | | |
| **Date ®** |  |  |  |  |  |
| Circle the appropriate answer | YES/NO | YES/NO | YES/NO | YES/NO | ANO/NE |
| If NO, how many reps? |  |  |  |  |  |
