# Supplementary material for: Enhancing Adherence to Home-Based Expiratory Muscle Strength Training in Parkinson Disease: Randomized Controlled Trial of an mHealth Intervention
Source: J Med Internet Res. 2026 Mar 11;28:e78022. doi: 10.2196/78022 (PMC12978541; doi:10.2196/78022)
Supplement: Multimedia Appendix 4 [file jmir-v28-e78022-s004.docx]

**Supplementary Table 3. Linear mixed-effects model for adherence**

| **Predictor** | **Estimate (β)** | **SE** | **z** | **p** | **95% CI** |
| --- | --- | --- | --- | --- | --- |
| Intercept | 942.000 | 92.925 | 10.137 | 0.000 | 759.870, 1124.130 |
| group[T.EG] | 50.316 | 131.416 | 0.383 | 0.702 | −207.254, 307.886 |
| interval[T.W8-24] | -416.835 | 132.143 | -3.154 | 0.002 | −675.831, −157.839 |
| seheps_group[T.≥59] | 27.615 | 145.793 | 0.189 | 0.850 | −258.133, 313.364 |
| group[T.EG]:interval[T.W8-24] | 496.985 | 186.880 | 2.659 | 0.008 | 130.707, 863.263 |
| group[T.EG]:seheps_group[T.≥59] | -15.931 | 200.361 | -0.080 | 0.937 | −408.632, 376.770 |
| interval[T.W8-24]:seheps_group[T.≥59] | 567.142 | 206.279 | 2.749 | 0.006 | 162.843, 971.441 |
| group[T.EG]:interval[T.W8-24]:seheps_group[T.≥59] | -426.482 | 286.193 | -1.490 | 0.136 | −987.410, 134.445 |
| Group Var | 7809.120 | 53.137 |  |  |  |

**Model information:** MixedLM (ML). Dependent variable: adherence. N (observations) = 126; Participants (groups) = 67; Log-likelihood = −940.2236; Scale = 167,658.2308; Min/Max group size = 1/2; Mean group size = 1.9.
**Random effects:** Random intercept (participant/ID) variance = 10,032.783.
**Reference levels:** group = Control; interval = W0–8; SEHEPS group = <59.

**Abbreviations and coding.**
EG = Experimental group (SpiroGym-assisted EMST); CG = Control group (conventional EMST).
W0–8 = Intensive phase (weeks 0–8); W8–24 = Maintenance phase (weeks 8–24).
SEHEPS = Self-Efficacy for Home Exercise Program Scale; SEHEPS ≥59 = not at risk for non-adherence; SEHEPS <59 = at risk.
β = Fixed-effect coefficient (estimate); SE = Standard error; z = Wald z-statistic; p = Two-sided p-value; 95% CI = 95% confidence interval.
MixedLM = Linear mixed-effects model; ML = Maximum likelihood estimation.
