# Supplementary material for: Enhancing Adherence to Home-Based Expiratory Muscle Strength Training in Parkinson Disease: Randomized Controlled Trial of an mHealth Intervention
Source: J Med Internet Res. 2026 Mar 11;28:e78022. doi: 10.2196/78022 (PMC12978541; doi:10.2196/78022)
Supplement: Multimedia Appendix 5 [file jmir-v28-e78022-s005.docx]

**Supplementary Table 4.** Four-week interval adherence analysis in patients without risk of non-adherence

| **Time interval** | **Group** | **n** | **Mean** | **SD** | **95% CI** | **p-value** | **Cohen d [95% CI]** |
| --- | --- | --- | --- | --- | --- | --- | --- |
| **W8-12** | CG | 12 | 288 | 139 | [209, 367] | 0.27 | 0.257  [-0.51, 1.02] |
|  | EG | 13 | 321 | 105 | [264,378] |  |  |
| **W12-16** | CG | 12 | 279 | 149 | [195,363] | 0.27 | 0.267  [-0.49,1.03] |
|  | EG | 13 | 314 | 103 | [258,370] |  |  |
| **W16-20** | CG | 12 | 290 | 152 | [204, 373] | 0.29 | 0.1  [-0.66, 0.86] |
|  | EG | 13 | 303 | 87 | [135, 375] |  |  |
| **W20-24** | CG | 12 | 263 | 154 | [176, 350] | 0.48 | 0.18  [-0.58, 0.94] |
|  | EG | 13 | 287 | 105 | [230, 344] |  |  |

**Abbreviations:** CG: control group; CI: confidence interval; EG: experimental group; SD: standard deviation.
