# Supplementary material for: Enhancing Adherence to Home-Based Expiratory Muscle Strength Training in Parkinson Disease: Randomized Controlled Trial of an mHealth Intervention
Source: J Med Internet Res. 2026 Mar 11;28:e78022. doi: 10.2196/78022 (PMC12978541; doi:10.2196/78022)
Supplement: Multimedia Appendix 6 [file jmir-v28-e78022-s006.docx]

**SpiroGym User Manual**

During a correct performance of the expiratory manoeuvre with the expiratory handheld device, the exhalation valve opens, and the air flow increases the sound level detected by the microphone. Simultaneously, the application gives visual feedback on the mobile phone screen (see training screen below) in real time using a curve depicting the current sound level.

**Training screen**


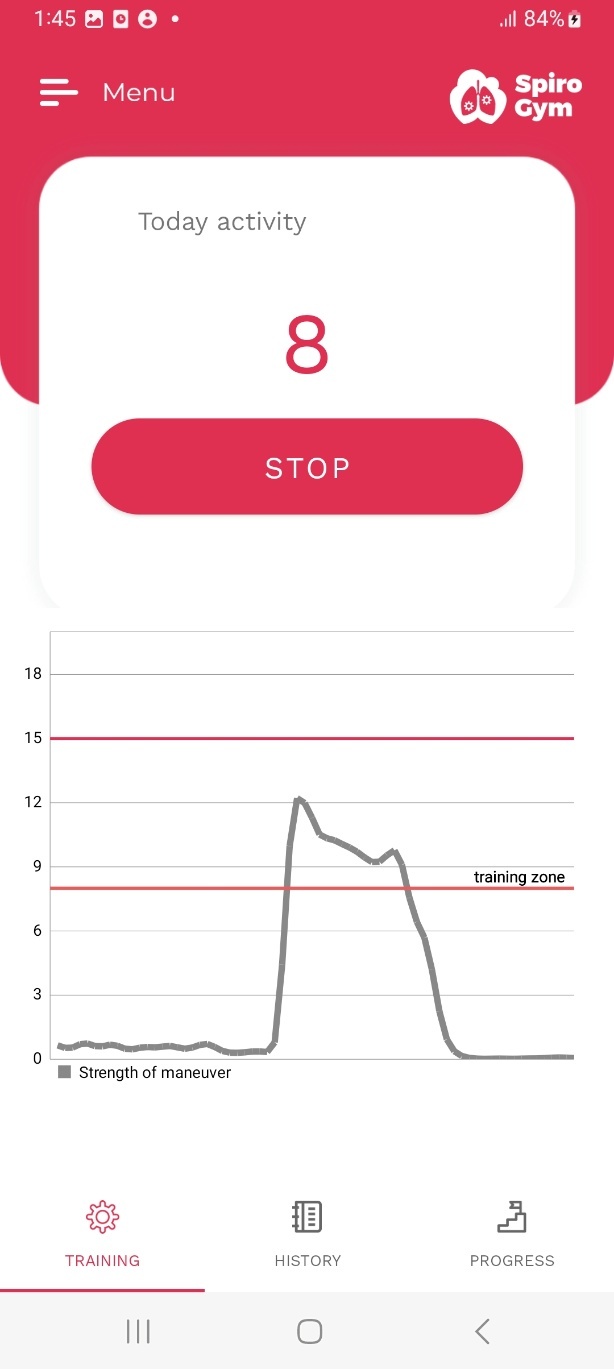


**Real time visual feedback**

The grey curve represents the noise generated by the expiratory trainer during forceful expiration.

Two horizontal lines represent the training zone set by the examiner. The patient’s expiratory effort is displayed as a grey graph. The goal is to reach as close as possible to the upper horizontal line..

**Maneuver completion indicator** Displays the number of maneuvers completed each day.

**History screen**


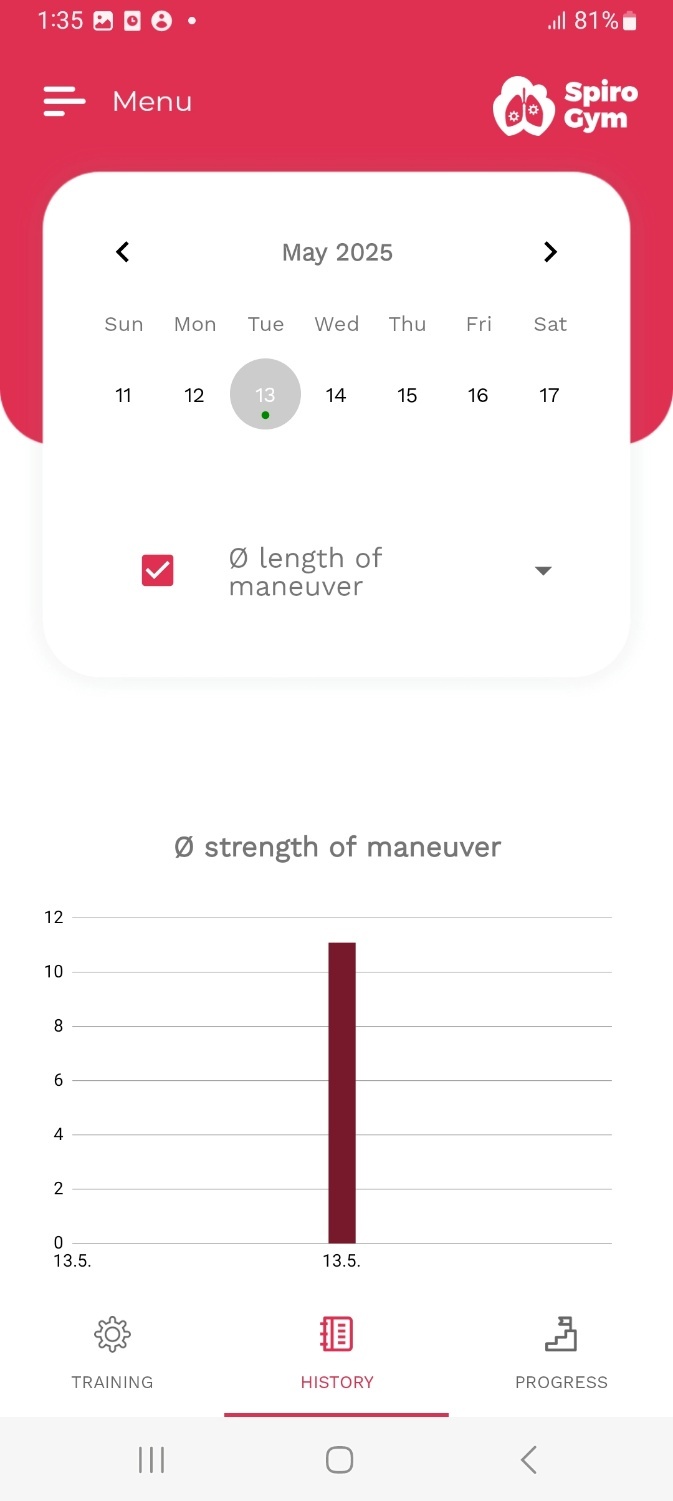


**SpiroGym E-Diaries**

An overview of completed training days and repetitions.

This section presents training data, including:

- Average maneuver duration
- Average maneuver strength
- Number of completed maneuvers

**Menu**


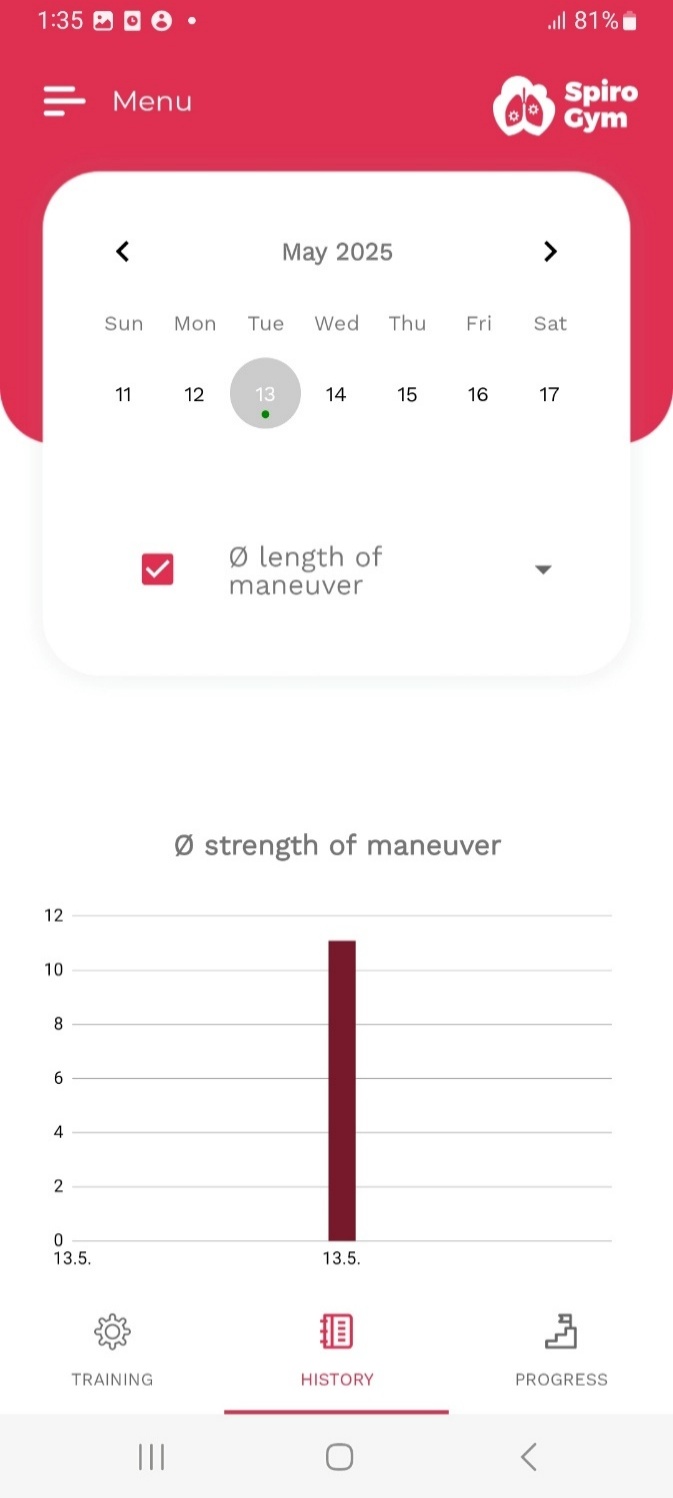


**Menu: Notifications**


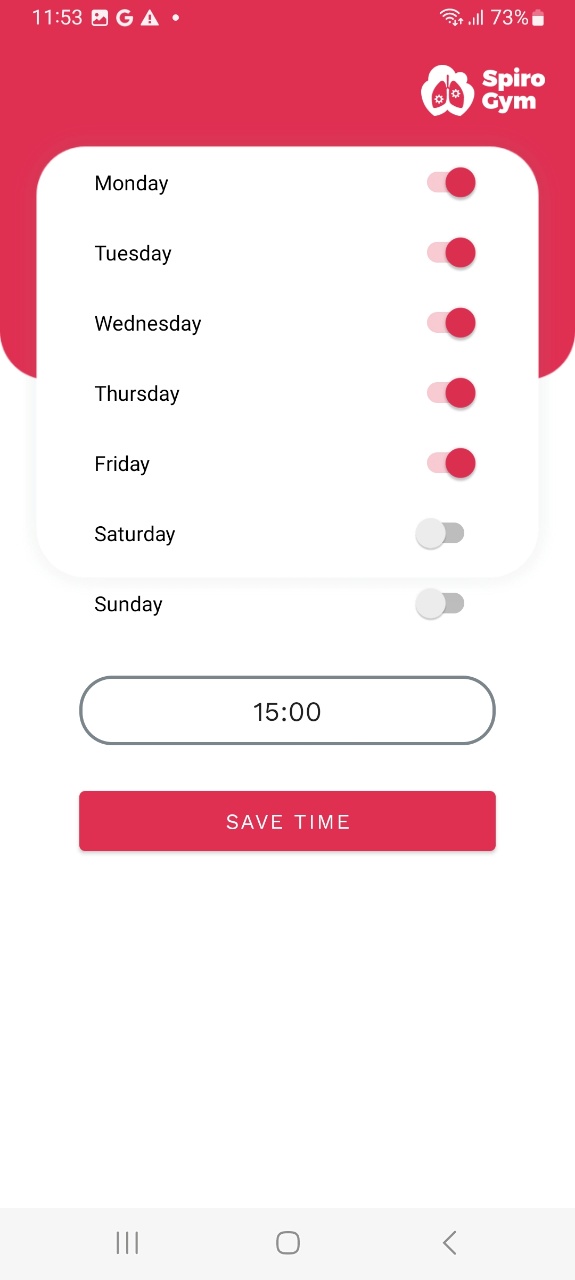


**Notifications**

Exercise notifications can be configured. The app issues reminders on selected days and times, indicating when training is scheduled.

**Menu: settings**


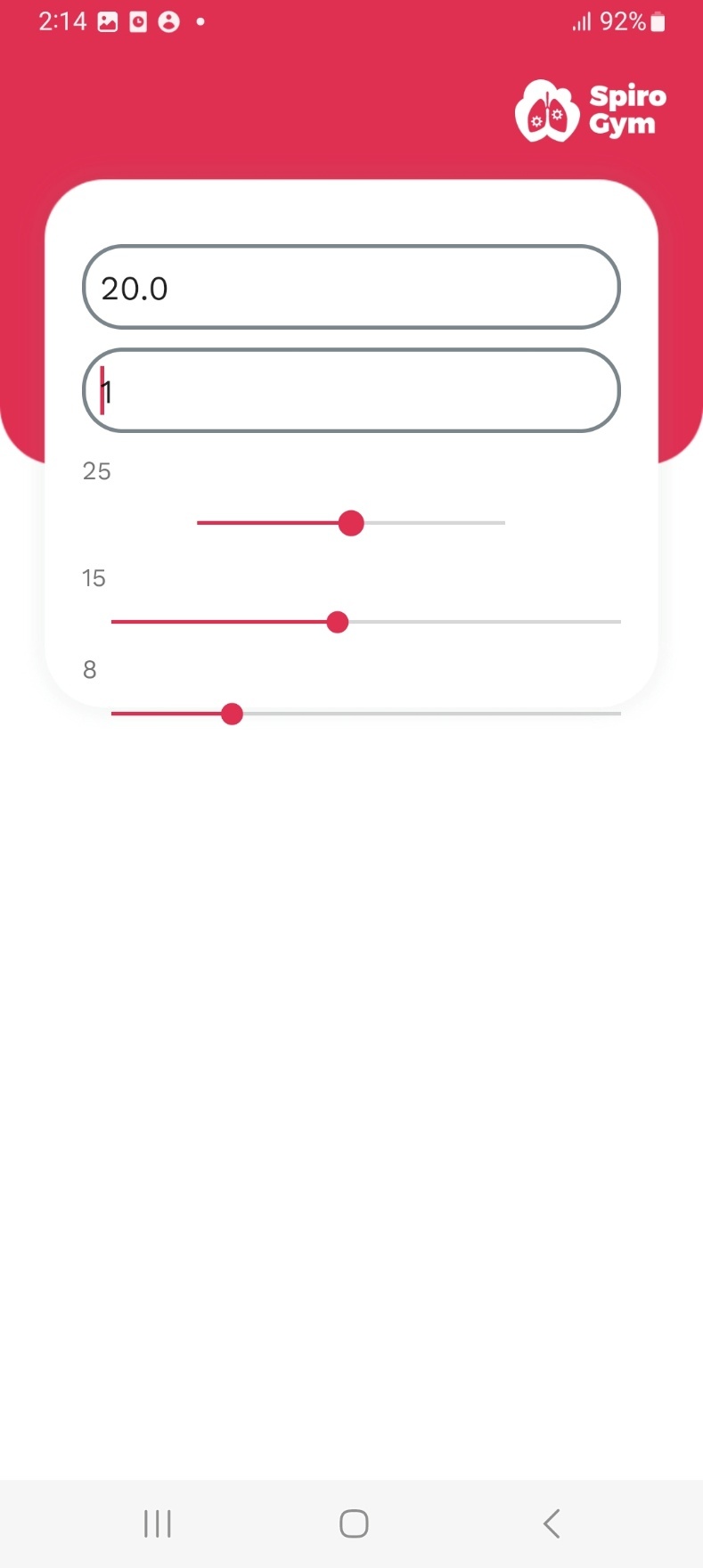


**Training zone position**: The position of the upper and lower horizontal lines in the graph can be adjusted.

Setting the recommended number of maneuvers for each training day.

**Microphone gain**: Adjustment (increase/decrease) of microphone sensitivity.

**Maximum Y-axis value**: Defines the upper limit of the graph's vertical axis in the visual feedback display.
